# Depression with Chronic Disease Is Associated with Increased Use of Medical Services and Medical Expenses in Hardcore Smokers

**DOI:** 10.3390/healthcare10081405

**Published:** 2022-07-27

**Authors:** Jeong-Won Han, Hanna Lee, Soyoon Min, Boyoung Lee

**Affiliations:** 1 College of Nursing Science, Kyung Hee University, Seoul 02447, Korea; hjw0721@khu.ac.kr; 2 Department of Nursing, Gangneung-Wonju National University, 150, Namwon-ro, Heungeop-myeon, Wonju-si 26403, Korea; 3 Department of Nursing, Graduate School, Kyung Hee University, Seoul 02447, Korea; papaugini2@naver.com (S.M.); soribit15@naver.com (B.L.)

**Keywords:** chronic disease, depression, health care costs, hardcore smokers, two part model

## Abstract

We aimed to investigate the association of chronic disease and depression with medical service use and expenses in hardcore smokers and provide basic data for health management system of hardcore smokers. This was a secondary data study involving 1735 smokers. Propensity score matching (PSM) was conducted to match hardcore smokers with regular smokers, and a two part model (TPM) was used based on the matched groups. In the case of general smokers, subjects with both depression and chronic disease had a significant relation to medical service use. In the case of hardcore smokers, subjects without depression and with chronic disease or with both depression and chronic disease had increased the use of medical services. The depression and chronic disease of general smokers did not affect the use of medical services. In the case of hardcore smokers, subjects who do not have depression and have only chronic disease (β = 0.20, *p* = 0.002) or with depression and chronic disease (β = 0.20, *p* = 0.014) significantly related the use of medical services. Conclusion: It is necessary to establish a health management system that considers both emotional states and chronic disease for hardcore smokers.

## 1. Introduction

The smoking epidemic is one of the greatest public health threats worldwide, causing more than eight million death annually [1]. Smoking is a known cause of cancer in almost all parts of the body, and leads to various diseases and premature mortality [2]. Smokers are more vulnerable to the recent epidemic of coronavirus disease 2019 (COVID-19) that infects the respiratory tract. The United States Centers for Disease Control and Prevention (CDC) classifies smokers as high-risk groups for COVID-19 infection [3,4]. Smoking is a direct and indirect social and economic burden to the country, not only due to medical expenses for disease treatment but also to productivity loss from disease treatment and premature mortality [5]. In Korea, approximately 35,000 individuals die from smoking-related diseases every year with an annual medical expense of approximately three trillion won [6]. Thus, to control the cost of medical utilities for smokers, alternative strategies must be sought to identify and approach the health care characteristics and use of smokers. 

To lower the smoking rate, various policies on smoking at huge costs have been introduced, and the status and cause of smoking have been analyzed in many countries. This has led to the concept of “hardening”, and that “hardened” smokers would pose a greater challenge for further reduction of smoking prevalence [7]. The term “hardcore smokers” has been defined differently by various groups with no clear consensus on the definition. In general, the term refers to smokers who have never quit in the past and are unwilling to quit smoking [8]. The criteria for this definition may include behaviors of smoking more than 15 cigarettes daily or long-term smoking of more than 5 years, which may help assess nicotine dependence [9,10]. As hardcore smokers are highly dependent on nicotine, these individuals are directly associated to various health problems. In particular, smoking is one of the most important factors that adversely affect and cause chronic diseases such as cardiovascular diseases, respiratory diseases, malignant tumors, cerebrovascular diseases, and mental diseases [4]. Therefore, hardcore smokers may cause an increase in medical service use and medical expenses, and it is essential to identify factors related to medical use and expenses to seek for countermeasures.

Smoking is also closely related to psychological problems. In a previous study, the rate of smokers was higher in those with mental health problems than in those without [11]. In addition, Weinberger et al. [12] reported that individuals with higher levels of depression smoke more and were highly likely to fail smoking cessation than those with lower levels of depression. Moreover, depression may lead to greater difficulties in smoking cessation, and withdrawal from smoking may aggravate depression in those suffering from depression [13]. As a result, depression is a factor that increases the use and expense of medical services, and combination of depression and smoking further increases such use and expenses. Therefore, in relation to the use of medical services by hardcore smokers, it is necessary to consider the decrease in physical function related to chronic disease as well as depression. Previous findings showed that chronic disease and depression increased medical expenses [14]. Moreover, about 12% of Korean adults suffer from depression and chronic disease. It has been shown to have a concomitant pattern of the more women, the higher the age, the higher the socioeconomic level, the lower the number, the higher the comorbidity of the complex disease. In the Korean Welfare Panel Survey [15], among 17,984 Korean adults, 11.8% suffer from both depression and chronic disease, and these individuals showed higher rates of outpatient treatment and hospital admission. In those with only depression, the number of outpatient treatment was not significantly different compared to those with depression and chronic disease, and those with depression were not hospitalized in the past year. However, studies on the use of medical services for people with depression and chronic diseases are limited. In particular, smokers need to consider the high-risk group that can accompany mental and physical diseases [14]. Accordingly, to assess the use of medical services and expenses in smokers, the mental and physical diseases must be also considered. Therefore, we aimed to investigate the association of chronic disease and depression with medical service use and medical expenses in hardcore smokers, and provide basic data for health management system of hardcore smokers.

## 2. Materials and Methods

### 2.1. Participants

The participants of this study were the 1735 current smokers among 6029 households that participated in the Korea Welfare Panel Study.

### 2.2. Measurement

#### 2.2.1. Hardcore Smokers

Hardcore smokers were defined as those who were current smokers aged <26 years, and were not defined to have fixed smoking behaviors [10]. In addition, those who smoked more than 15 cigarettes per day, had not tried to quit smoking in the past year, and had no plans to quit smoking within the next 6 months, were selected [9]. Those who did not satisfy the criteria of hardcore smokers were considered regular smokers.

#### 2.2.2. Propensity Score Matching

Propensity score matching (PSM) variables were defined as variables related to hardcore smokers and medical service use: sex (male, female), age, education level (under middle school, high school graduate, professional university or higher), personal income (quintile income), marital status (married or single living with spouse) [16], alcohol consumption (never, less than once a month, 2–4 times a month, 2–3 times a week, more than 4 times a week), and occupation type (wage or non-wage workers) [17].

#### 2.2.3. Depression

Depression was evaluated using Center for Epidemiologic Studies Depression Scale (CES-D), which was used in the Welfare Panel Survey [18]. CES-D consisted a total of 11 items, which were evaluated on a 4-point scale, and a higher sum of the scores indicated greater severity of depression. In addition, the total score of CES-D was multiplied by 20/11. If the calculated value was ≥16, the participant was considered “not depressed” and “depressed”, respectively [15].

#### 2.2.4. Chronic Diseases

Those who were prescribed medications for treatment of 32 chronic diseases among the items of the Welfare Panel Survey were considered to have a chronic disease. Moreover, those who had chronic diseases but were not prescribed medications due to economic circumstances were included.

#### 2.2.5. Use of Medical Services

For medical service use, outpatient or inpatient treatment in the last year was evaluated. Those who had not received treatment were considered to have “not used medical services” and others who had received treatment for more than once were considered to have “used medical service”.

#### 2.2.6. Medical Expense

The average monthly medical expenses of those who have received outpatient or inpatient treatment in the past year were assessed. Medical expenses defined by the Welfare Panel included admission fees, outpatient treatment expense, dental treatment expense, surgery expense (implants, plastic surgery), medication expense, nursing expense, postpartum care expense, health examination expanse, health supplement expense, and health care supplies (glasses, contact lenses, etc.) paid by the participants.

### 2.3. Data Collection and Analysis

Data available to the public on the Korea Welfare Panel Survey website (https://www.koweps.re.kr:442/main.do, accessed on 17 December 2021) was downloaded and used in this study. The disclosed data did not include information that could be used for personal identification of the participants. The data were analyzed using SPSS window 23.0 version (Data solution Inc., Korea) and R project 4.1 version [19].

Participant characteristics were analyzed by calculating the frequency/percentage and mean/standard deviation. PSM was conducted to match hardcore smokers with regular smokers. To conduct PSM, experimental (hardcore smokers) and control (regular smokers) groups were set. Propensity score (PS) was calculated through logistic regression using PSM variables. Minimum distance method was used to pair PS of the experimental and control groups in 1:1 ratio. Standardized mean difference and PS distribution were assessed to analyze the results of matching, and cross-analysis was conducted to evaluate the differences between the matching variables before and after matching. To determine the effects of depression and chronic disease on medical service use and medical expenses in matched regular and hardcore smokers based on the PSM results, a two part model (TPM) was used. In the first step of TPM analysis, logistic regression analysis was conducted by controlling for PSM variables, health insurance subscription type (employee or local insurance), and private insurance subscription. In the second step, logistic regression analysis was conducted after selecting who have used medical services in the past year and controlling for PSM variables, health insurance subscription type, and private insurance subscription. The distribution of medical expenses was not normally distributed and showed significant differences depending on the number of medical service use. Thus, natural log was used to assess the distribution of medical expenses [20]. 

This study was approved for review exemption (GWNUIRB-R2021-86) by the Institutional Review Board as a secondary data analysis.

## 3. Results

### 3.1. Participant Characteristics

A total of 1735 participated in the study before matching and the general characteristics are described in Table 1. 

### 3.2. Distribution of Propensity Score before and after Matching

**Figure 1:**
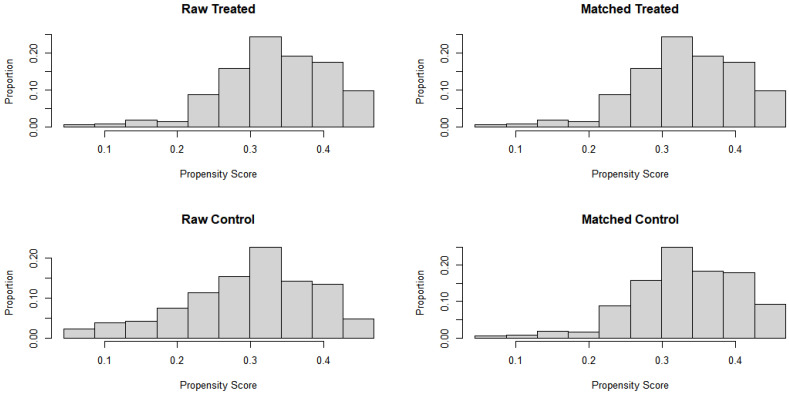

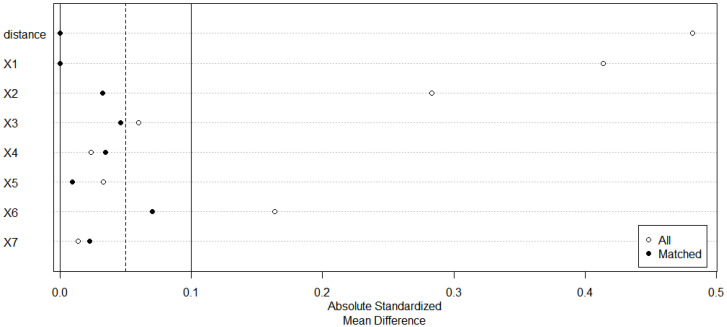
Data balance after propensity score matching. X1 = Gender, X2 = Age, X3 = Education level, X4 = Personal income, X5 = Marital status, X6 = Alcohol consumption, X7 = Working type.

Figure 1 present the distribution of PS after matching regular and hardcore smokers by calculating the PS using PS matching variables (sex, age, education level, personal income, marital status, alcohol consumption, and occupation type). The total distance after matching was close to 0.0 compared to the total distance of 0.5 before matching. For all matching variables, the standard mean difference of the variables between hardcore and regular smokers following PSM were closer to 0 after matching (Table 2).

### 3.3. Comparison of Characteristics between Groups before and after Matching

Cross-analysis between the groups before matching showed significant differences in sex (χ^2^ = 23.34, *p* < 0.001), age (χ^2^ = 40.28, *p* < 0.001), and alcohol consumption (χ^2^ = 66.93, *p* < 0.001); however, after matching, there were no significant differences observed in all variables between the groups (Table 3).

### 3.4. Association of Depression and Chronic Disease on Medical Service Use and Medical Expense in Hardcore Smokers

Table 4 shows the level of depression, medical service use, and expense of the subjects according to the presence or absence of depression and chronic disease before TPM. To use TPM on the matched groups, first, matching variables as well as health insurance subscription type and private insurance were set as covariates. Logistic regression analysis was conducted to determine whether depression and chronic disease associated with medical service use as shown in Table 5. In general smokers, depression and chronic disease had 1.13–6.18 times more significant effects on medical service use. In hardcore smokers, Without depression and with chronic disease and depression with chronic disease had increased 1.77–14.78, 1.41–12.08 times more on the use of medical services.

In the second step, multiple regression analysis was conducted to evaluate the effects of depression and chronic disease on medical service use in smokers with experiences of using medical services. In regular smokers, depression and chronic disease did not have effects on the use of medical services. In contrast, in hardcore smokers, Without depression and with chronic disease (β = 0.20, *p* = 0.002) and depression with chronic disease (β = 0.20, *p* = 0.014) significantly affected the use of medical services.

## 4. Discussion

This study investigated the association between depression and chronic disease on medical service use and medical expenses in hardcore smokers in Korea. Based on the results of this study, the implications are discussed as follows.

Our findings showed that in regular smokers, medical service use was significantly related only by depression with chronic disease. Meanwhile, in hardcore smokers, both chronic disease with and without depression had significant effects on the use of medical services. This finding supported the findings of a previous study [15] in which analysis of the patterns of chronic disease and medical service use in adults showed that depression with chronic disease increased the number of outpatient visits and admission rate. In addition, depression and chronic disease have been suggested to be closely related [21]. As physical and psychological symptoms interact and affect the functional state of patients, patients with both chronic diseases and depression are more likely to use medical services. Moreover, Park et al. [9] previously reported that hardcore smokers have a higher rate of depression and experienced extreme stress more frequently than regular smokers, suggesting that depression with smoking has significant effects on the use of medical services. Such depression and chronic diseases require long-term treatments. These increase the burden of support for both the participants as well as the nation in terms of financial aspect [22]. Thus, active interventions are needed. In particular, depression with chronic diseases were related to the use of medical services in both hardcore and general smokers. This suggests that mental health interventions are required for all types of smokers with chronic diseases.

There was no significant association between depression without chronic disease and use of medical services between general and hardcore smokers in our study. This reflects the general lack of positive perception about the use of medical services for mental health problems in Korea than in other countries [23]. In particular, it is thought that hardcore smokers choose to smoke to cope with psychological challenges, which leads to reduced use of medical services. However, depression is a major outpatient disease among different mental diseases and can be managed with continuous medication treatment. Additionally, previous findings showed that hardcore smokers tend to smoke as a coping mechanism against depression [11], suggesting that early assessment of depression in hardcore smokers need to be reflected in smoking-related policies. This is essential to prevent the symptoms and functional deterioration experienced by hardcore smokers as psychological mechanisms affect mechanical mechanisms. Thus, active policy interventions would be necessary.

In regular smokers, depression and chronic disease did not relate to medical expenses. In contrast, in hardcore smokers, chronic disease with and without depression had significantly associated with medical expenses. This may be related to the medical expenses caused by various health problems in hardcore smokers, who smoked frequently compared to general smokers. Although there is a lack of studies that compared and analyzed the medical expenses of regular smokers and hardcore smokers, one study simulated smoking, life expectancy, and chronic disease in American smokers [24]. In that score, smoking cessation increased life expectancy in all smokers, especially in hardcore smokers, suggesting that interventions for smoking cessation in hardcore smokers may be effective in reducing the medical expenses. In particular, smoking cessation in regular smokers increase the survival rate by 0.28% of over the age of 65 adult in Korea, 2.17% in the U.S, and 1.00% in Singapore. In contrast, in hardcore smokers, smoking cessation increased the survival rate by 1.51% in adults aged >65 years in Korea, 4.69% in the U.S, and 9.00% in Singapore, indicating that smoking cessation in hardcore smokers may lead to several health benefits. 

Therefore, government and local communities need to develop financial support and education programs for hardcore smokers and subsequently prevent regular smokers from becoming one. However, unlike hardcore smokers, regular smokers did not relate to medical expenses. Although smoking does not immediately lead to onset of diseases and poor health [22], lung cancer may occur after 20–30 years [25]. Thus, delayed health effects of smoking may explain the lack of relationships by regular smokers on medical expenses compared to that by hardcore smokers. In previous studies, smokers tend to quit smoking after the onset of diseases. In a study of adults aged >18 years in China, more than 50% of smokers stopped smoking at an average of 8 years after diagnosis of cerebral apoplexy, diabetes, hypertension, cancer and coronary diseases. The mean time required to quit smoking was greater in those diagnosed with chronic obstructive pulmonary disease, chronic gastritis, chronic bronchitis, and asthma [26]. These findings show that smoking cessations requires a long period of time despite the personal efforts of smokers, and that smoking leads to enormous medical expenses. A longer period and higher frequency of smoking lead to greater adverse effects on health. Therefore, it is necessary to create an environment to promote smoking cessation even in the absence of health problems, and programs, campaigns, and repeated education related to smoking cessation must be provided. The government promotes community-centered smoking cessation education, counseling, and treatment. In particular, local governments and educational institutions such as middle, high school, universities, workplaces, and private organizations will establish local smoking cessation programs. In addition, anti-smoking publicity and education for the entire nation will be strengthened. Promote a segmented public relations strategy for each target audience. The Non-smoking areas should be expanded from the present and education to prevent secondhand smoke should be strengthened.

## 5. Conclusions

This study determined the effects of depression and chronic disease on the use of medical services and medical expenses in hardcore smokers, using the data from the Korea Welfare Panel Survey. This study is relevant in that objectively quantified national data was used to determine the effects on medical service use and medical expenses in consideration of not only sociodemographic characteristics and smoking-related factors but also health-related lifestyle habits. In addition, this study is significant as differences between regular and hardcore smokers, which were not evaluated in previous studies, were investigated. To reduce the social and economic costs of smoking, social health awareness need to be increased through smoking-related education and expansion of smoking cessation policies in the future. However, several limitations must be considered in interpreting the results of this study. First, only patient expenses were analyzed. Thus, the total medical expenses may be different. In future studies, data linked with health insurance would be necessary for a more accurate analysis. Second, this study conducted cross-sectional data analysis and was limited in investigating the causal relationships. The depression scale used in this study is CES-D, which is used as a depression scale in epidemiologic studies. In a future study, it is also necessary to confirm the medical expenses and medical use in the relationship between chronic disease and depression by using a symptom-centered measurement of depression. In addition, it is necessary to confirm the relationship between the severity of chronic disease and the degree of depression on the use of medical services. 

## Figures and Tables

**Table 1:** General characteristics of study subjects before matching (*n* = 1735).

Variables	Category	*n*	%	M ± SD
Gender	Male	1598	92.1	
	Female	137	7.9	
Age (year)	19–29	189	10.9	50.66 ± 15.70
	30–39	233	13.4	
	40–49	430	24.8	
	50–59	388	22.4	
	>60	495	28.5	
Education level	Under middle school	197	11.4	
	High school	165	9.5	
	Over college	1373	79.1	
Personal income (year)	1st quartile	160	9.2	6223.97 ± 4720.96
	2nd quartile	324	18.7	
	3rd quartile	249	14.4	
	4th quartile	325	18.7	
	5th quartile	677	39.0	
Marital status	Married & spouse cohabitation	868	50.0	
	Single or spouse non-cohabitation	867	50.0	
Alcohol consumption	None	134	7.7	
	Less than once month	378	21.8	
	2–4 times a month	466	26.9	
	2–3 times a week	309	17.8	
	4 or more times a week	448	25.8	
Working type	Wage earner	952	54.9	
	Self-employed worker	783	45.1	
National Health insurance	Employee’s health insurance	1085	62.5	
	Local health insurance	536	30.9	
	Other	114	6.6	
Private health insurance	None	449	25.9	
	≥1	1286	74.1	
Smoker type	General smoker	1202	69.3	
	Hardcore smoker	533	30.7	
Medical service experience	None	256	14.8	
	≥1	1479	85.2	

M = Mean, SD = Standard deviation.

**Table 2:** Data balance after propensity score matching.

Variables	Before Propensity Score Matching			After Propensity Score Matching		
	Hardcore Smoker (*n* = 533)	General Smoker (*n* = 1202)	Standardized Mean Difference	Hardcore Smoker (*n* = 533)	General Smoker (*n* = 533)	Standardized Mean Difference
Gender	1.03	1.10	−0.41	1.03	1.03	0.00
Age (year)	3.66	3.34	0.28	3.66	3.70	−0.03
Education level	2.64	2.69	−0.06	2.64	2.68	−0.04
Personal income (year)	3.57	3.60	−0.02	3.57	3.52	0.03
Marital status	1.95	1.95	0.03	1.95	1.95	−0.01
Alcohol consumption	3.46	3.26	0.16	3.46	3.37	0.07
Working type	1.44	1.45	−0.01	1.44	1.45	0.02

**Table 3:** Homogeneity of subjects’ characteristics.

Variables	Before Matching (*n* = 1735)				χ^2^ (*p*)	After Matching (*n* = 1066)				χ^2^ (*p*)
Category	Hardcore Smoker’s Group (*n* = 533)		General Smoker’s Group (*n* = 1202)			Hardcore Smoker’s Group (*n* = 533)		General Smoker’s Group (*n* = 533)		
	*n*	%	*n*	%		*n*	%	*n*	%	
Gender					23.34 (<0.001)					0.00 (1.00)
Male	517	29.8	1081	62.8		517	48.5	517	48.5	
Female	16	0.9	121	7.0		16	1.5	16	1.5	
Age										
19–29	22	1.3	167	9.6	40.28 (<0.001)	22	2.1	28	2.6	2.38 (0.665)
30–39	69	4.0	164	9.5		69	6.5	56	5.3	
40–49	136	7.8	294	16.9		136	12.8	134	12.6	
50–59	142	8.2	246	14.2		142	13.3	141	13.2	
>60	164	9.5	331	19.1		164	15.4	174	16.3	
Education level										
Under middle school	65	3.7	132	7.6	2.01 (0.366)	65	6.1	58	5.4	0.60 (0.741)
High school	57	3.3	108	6.2		57	5.3	54	5.1	
Over college	411	23.7	962	55.4		411	38.6	421	39.5	
Personal income										
1st quartile	42	2.4	118	6.8	6.66 (0.155)	42	3.9	55	5.2	4.21 (0.377)
2nd quartile	103	5.9	221	12.7		103	9.7	108	10.1	
3rd quartile	91	5.2	158	9.1		91	8.5	72	6.8	
4th quartile	101	5.8	224	12.9		101	9.5	97	9.1	
5th quartile	196	11.3	481	27.7		196	18.4	201	18.9	
Marital status										
Married & spouse cohabitation	328	18.9	540	31.1	0.37 (0.542)	328	30.8	322	30.2	0.14 (0.706)
Single or spouse non-cohabitation	205	11.8	662	38.2		205	19.2	211	19.8	
Alcohol consumption										
None	124	7.1	95	5.5	66.93 (<0.001)	124	11.6	127	11.9	4.68 (0.321)
Less than once month	39	2.2	305	17.6		39	3.7	44	4.1	
2–4 times a month	73	4.2	316	18.2		73	6.8	85	8.0	
2–3 times a week	150	8.6	162	9.3		150	14.1	159	14.9	
4 or more times a week	147	8.5	324	18.7		147	13.8	118	11.1	
Working type										
Wage earner	295	17.0	657	37.9	0.07 (0.790)	295	27.7	301	28.2	0.13 (0.711)
Self-employed worker	238	13.7	545	31.4		238	22.3	232	21.8	

**Table 4:** Degree of subject’s depression. chronic disease, medical service use and medical cost.

Groups	*n*	Depression (Past 2 Week)		Use of Medical Services						Medical Expense (Number/Month) (Unit: Ten Thousand Won: KRW)	
				Total (Number/Year)		Outpatient (Number/Year)		Inpatient (Number/Year)			
		Mean	S.E	Mean	S.E	Mean	S.E	Mean	S.E	Mean	S.E
Group 1	204	16.36	0.01	2.59	0.36	2.57	0.36	0.02	0.01	16.08	1.53
Group 2	123	16.36	0.01	15.11	1.13	14.93	1.13	0.17	0.04	24.45	2.34
Group 3	330	33.36	3.09	3.56	0.62	3.51	0.61	0.06	0.01	15.28	1.20
Group 4	404	33.36	2.83	20.75	1.42	20.61	1.42	0.14	0.02	19.34	1.26

SE = Standard error. Group 1 = Without depression and without chronic disease, Group 2 = Without depression and with chronic disease, Group 3 = With depression and without chronic disease.

**Table 5:** Effect of subject’s depression and chronic disease on medical service use and medical cost.

	Part 1: Use of Medical Services General Smoker = 533 Hardcore Smoker = 533						Part 2: Medical Cost General Smoker = 444 Hardcore Smoker = 387					
Variables	B	S.E	*p*	Exp(B)	95% CI of Exp(B)		B	S.E	β	*p*	95% CI of Exp(B)	
					Lower	Upper					Lower	Upper
General smoker												
Gender	−1.22	1.16	0.294	0.29	0.03	2.88	−0.31	0.28	−0.06	0.253	−0.61	0.49
Age	−0.01	0.01	0.903	0.99	0.97	1.02	0.01	0.10	0.15	0.033	−0.05	0.35
Education level	0.30	0.47	0.523	1.35	0.54	3.41	−0.12	0.16	−0.05	0.431	−0.37	0.26
Personal income	0.32	0.23	0.177	1.38	0.86	2.20	0.49	0.30	0.30	<.001	−0.29	0.89
Marital status	0.27	0.31	0.400	1.30	0.70	2.24	0.49	0.14	0.20	<.001	−0.08	0.48
Alcohol consumption	0.41	0.29	0.170	1.50	0.84	2.67	−0.31	0.12	−0.12	0.011	−0.36	0.12
Working type	−0.79	0.35	0.024	0.45	0.022	0.90	−0.13	0.14	−0.06	0.338	−0.34	0.22
National Health insurance	0.56	0.31	0.078	1.75	0.93	3.26	0.14	0.13	0.06	0.255	−0.20	0.32
Private health insurance	−0.28	0.37	0.474	0.76	0.36	1.59	0.01	0.15	0.02	0.979	−0.27	0.31
Without depression and without chronic disease	1						0					
Without depression and with chronic disease	1.45	0.29	0.997	1.39	0.01	1.89	0.38	0.20	0.12	0.066	−0.27	0.51
With depression and without chronic disease	0.12	0.28	0.653	1.13	0.65	1.94	0.11	0.18	0.04	0.571	−0.31	0.39
With depression and with chronic disease	3.78	0.58	<.001	3.86	1.13	6.18	0.35	0.18	0.15	0.053	−0.20	0.50
Hardcore smoker												
Gender	0.46	1.03	0.655	1.58	0.21	11.95	0.50	0.30	0.08	0.099	−0.51	0.67
Age	0.01	0.01	0.440	1.01	0.98	1.04	0.01	0.10	0.11	0.124	−0.09	0.31
Education level	0.67	0.43	0.121	1.95	0.83	4.58	0.08	0.15	0.03	0.594	−0.26	0.32
Personal income	0.11	0.22	0.617	1.11	0.72	1.72	0.70	0.28	0.43	<0.001	−0.12	0.98
Marital status	0.62	0.28	0.028	1.86	1.06	3.26	0.20	0.14	0.08	0.148	−0.20	0.36
Alcohol consumption	−0.32	0.34	0.348	0.72	0.39	1.42	−0.11	0.13	−0.04	0.428	−0.30	0.22
Working type	0.14	0.29	0.627	1.15	0.65	2.04	−0.23	0.13	−0.10	0.074	−0.36	0.16
National Health insurance	−0.08	0.29	0.778	0.92	0.52	1.63	0.06	0.13	0.03	0.611	−0.23	0.29
Private health insurance	0.19	0.33	0.566	1.21	0.63	2.13	0.02	0.14	0.01	0.891	−0.27	0.29
Without depression and without chronic disease	1						0					
Without depression and with chronic disease	3.25	0.76	<0.001	5.73	1.77	14.78	0.65	0.10	0.20	0.002	0.01	0.40
With depression and without chronic disease	0.33	0.26	0.206	1.39	0.83	2.32	0.16	0.19	0.06	0.391	−0.31	0.43
With depression and with chronic disease	4.13	0.65	<0.001	6.21	1.41	12.08	0.36	0.08	0.16	0.044	0.01	0.32

SE = Standard error, 95% CI = 95% confidence interval.

## Data Availability

Not applicable.
